# Influencing factors and improvement methods of coronary artery plaque evaluation in CT

**DOI:** 10.3389/fcvm.2024.1395350

**Published:** 2024-06-25

**Authors:** Yaqi Gao, Yao Pan, Chongfu Jia

**Affiliations:** Department of Cardiovascular Radiology, The First Affiliated Hospital of Dalian Medical University, Dalian, China

**Keywords:** coronary, plaque, computed tomography, angiography, evaluation

## Abstract

Accurate evaluation of the nature and composition of coronary plaque involves clinical follow-up and prognosis. Coronary CT angiography is the most commonly non-invasive method for plaque evaluation, however, the qualitative and quantitative evaluation of plaque based on CT value is inaccurate, due to the influence of luminal attenuation, tube voltage, parameter setting and the subjectivity.

## 1 Introduction

Coronary heart disease (CHD) incidence has been on the rise year by year, with its mortality rate ranking first among cardiovascular diseases (1). The primary cause of cardiovascular events is the rupture of unstable coronary artery plaques followed by thrombus formation. Numerous postmortem and intravascular ultrasound studies have shown a close correlation between plaque nature and composition with stability, particularly emphasizing the heightened activity and instability of non-calcified plaques, especially those with high lipid content (2, 3). Moreover, the assessment of plaque burden and composition has a good predictive value for cardiovascular events, and can further evaluate clinical risk factors, reflect drug treatment and adverse clinical outcomes (4–6). Therefore, accurately assessing plaque nature and composition is crucial for risk stratification and prognosis evaluation in patients with CHD.

Intravascular imaging techniques such as optical coherence tomography (OCT) are considered the gold standard for assessing plaque nature and composition *in vivo*. However, their invasive, complexity of operation, and cost make them challenging for widespread clinical adoption (7). In recent years, coronary computed tomographic angiography (CCTA) has become a routine clinical tool for non-invasive evaluation of plaques (8). Nevertheless, challenges persist in accurately assessing coronary artery plaque nature and composition based on CT values due to factors such as luminal attenuation, tube voltage, parameter settings, and subjectivity, leading to significant overlap in CT values between fibrous and lipid-rich plaques (9). In this review, we summarize the methods and influencing factors of plaque assessment for CCTA, and provide the corresponding improvement methods.

### 1.1 Qualitative and quantitative assessment of plaques in CCTA

**Figure 1:**
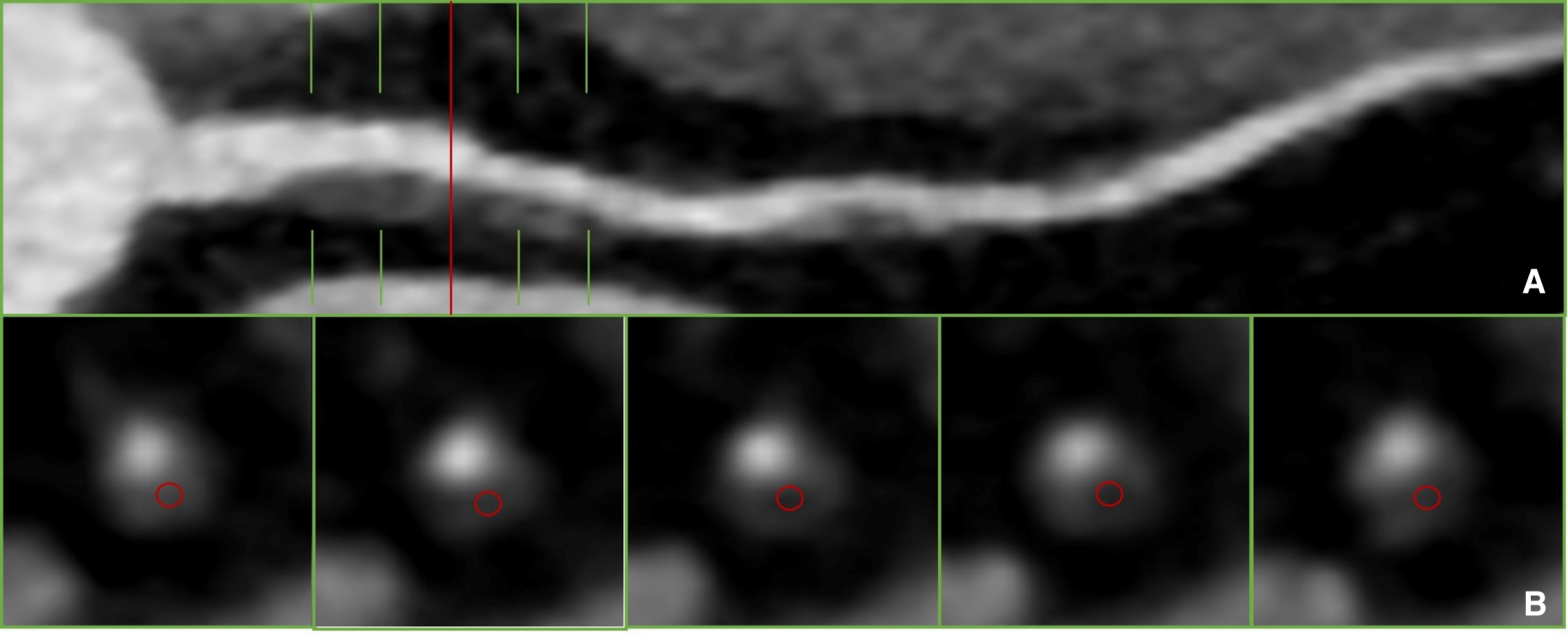
Plaque recognition and CT value measurement in previous literature (10). (**A**) Non-calcified plaque was detected on proximal segment of LAD on curved reformation. (**B**) The measurement of qualitative.

The qualitative assessment of plaques involves manually selecting regions of interest (ROIs) and determining plaque characteristics based on the measured CT values, as shown in Figure 1. However, unified standard for ROI selection are not clearly defined, including criteria such as the selection of representative slices, distance from the lumen, and ROI size. It has been reported that efforts should be made to avoid the lumen and vessel walls while encompassing the plaque as much as possible. Multiple ROIs are then measured, and their average is calculated (10). However, specific guidelines on how much to avoid are not clearly defined. Currently, to use a generalized HU-criterion is not yet possible as the reported HU values vary considerably (3, 11–16), as shown in Table 1. Matsumoto et al. (3) conducted CCTA examinations on 77 patients with known or suspected coronary artery disease, using intravascular ultrasound (IVUS) as the gold standard. They found that using 45 HU as the cutoff value resulted in higher diagnostic accuracy for lipid plaques. In another study, Han et al. (12) identified 75 HU as the optimal cutoff value for accurate identification of lipid plaques, showing good consistency with histological regions rich in lipid plaques. This variability in cutoff values poses challenges for widespread clinical application.

**Table 1:** Comparison of attenuation values used in selected studies.

Study	Year	Attenuation values (in HU units)		
		Lumen	Lipid	Fibrosis
Matsumoto et al. (3)	2019	193 (165–213) HU	<45 HU	≥45 HU
Matsumoto et al. (11)	2019	431.2 ± 67.0 HU	<58 HU	≥58 HU
Han et al. (12)	2018	444 (201–725) HU	<75 HU	≥75 HU
Yamaki et al. (13)	2012	326 ± 55 HU	<50 HU	50–170 HU
You et al. (14)	2016	NA	0–49 HU	50–129 HU
Wang et al. (15)	2018	276.05 ± 4.96 HU	<60 HU	60–200 HU
Takahashi et al. (16)	2016	357 ± 65 HU	<56 HU	56–210 HU

**Figure 2:**
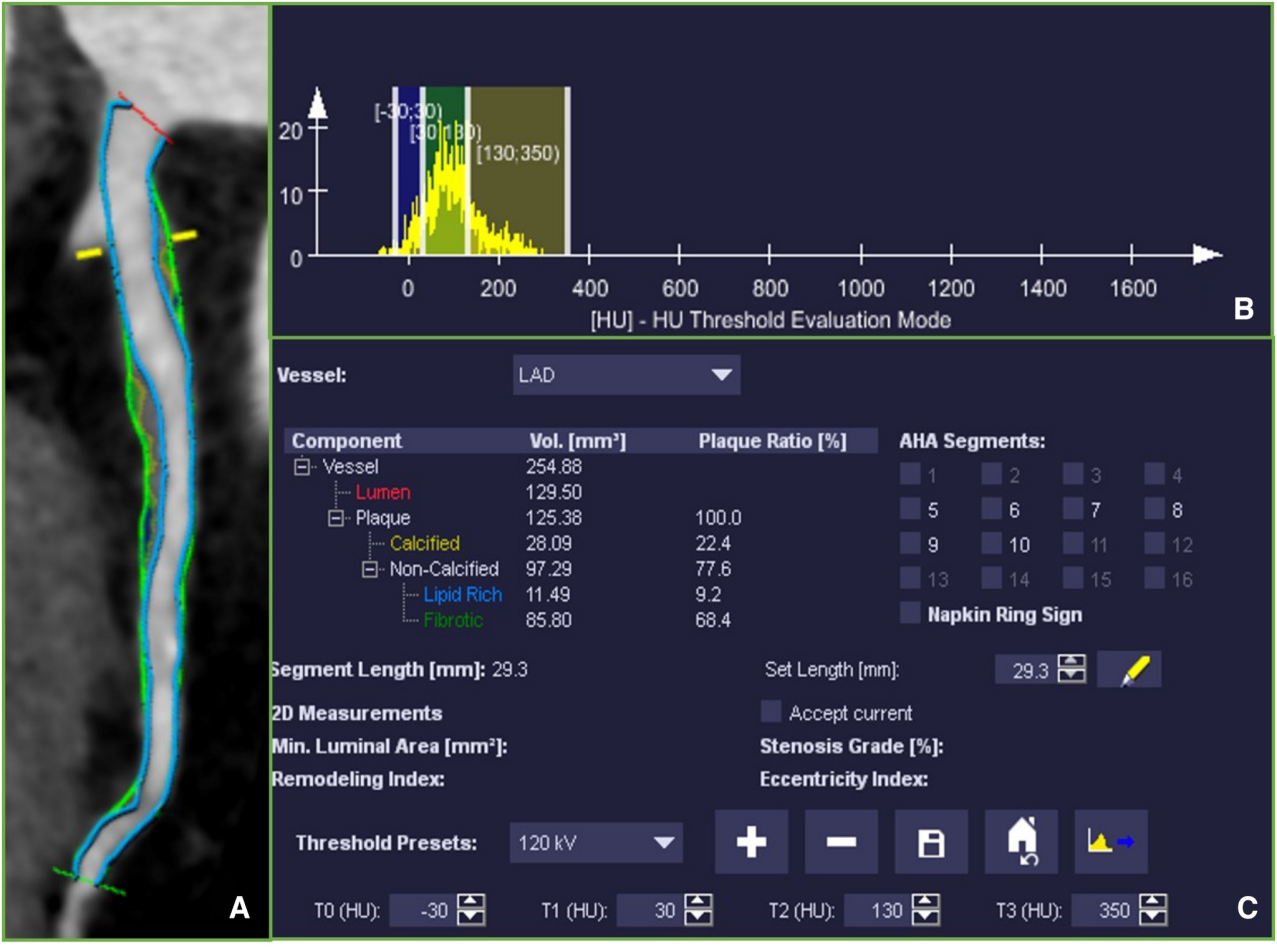
(**A**) Multiplanar reconstruction of the left anterior descending coronary artery (LAD) showing the whole coronary artery lesion, ranging from the proximal to distal lesion marker (green markers). Fibrotic tissue was labeled in dark yellow, Fibro-fatty tissue in light green and necrotic core was labeled in blue. (**B**) A histogram of the distribution of CT attenuation within the plaque. (**C**) Plaque volumes were derived for the quantitative analysis software based on pre-set thresholds.

In contrast to the qualitative assessment of plaque characteristics, the quantitative measurement of plaque components involves semi-automatically delineating the entire plaque using quantitative analysis software, as shown in Figure 2. Component volumes are obtained based on pre-set thresholds. Although this eliminates the subjectivity of manually selecting ROIs, there is still inconsistency in the definition of component thresholds. Most studies define −30 to 30 HU, 30–130 HU, 131–350 HU, and >350 HU as necrotic core, fibrofatty, fibrous, and calcified components, respectively (17). Some studies define −30 to 75 HU and 76–130 HU as lipid and fibrofatty components, respectively (18). Takx et al. (19) define fibrous plaque as 70–129 HU, not fibrofatty. Therefore, the cutoff values for distinguishing lipid and fibrous components remain a contentious issue. Furthermore, most studies define 131–350 HU as fibrous components, which significantly differs from the qualitative cutoff value for fibrous plaques. This definition contradicts even the definition in calcium scoring, where >130 HU is considered calcified plaque. Takahashi et al. (16) demonstrated that the fibrous component load measured by CCTA was poorly correlated with the gold standard virtual IVUS, with a correlation coefficient of only 0.18. This discrepancy may be attributed to the influence of partial volume effects in the vessel lumen, leading to the erroneous classification of 131–350 HU as fibrous plaques, which may not necessarily represent true fibrous tissue.

### 1.2 Factors influencing plaque evaluation

There are numerous factors affecting the assessment of coronary artery plaques based on CT, primarily including partial volume effect of lumen, imaging parameters, and subjective factors.

Firstly, luminal attenuation is the most significant influencing factor. This is due to the limited spatial resolution of CT equipment. Studies indicate a significant positive correlation between lipid and fibrous plaques and luminal HU, with correlation coefficients of 0.49 and 0.32, respectively. In contrast, calcified plaques show no correlation with luminal HU (*r* = 0.04) (11). Takagi et al. (20) further demonstrated that as luminal attenuation increases, the proportion of calcified components also increases, while the proportions of fibrous plaques, fibrous lipid, and necrotic core decrease. Kristanto et al. (21) using a phantom model, investigated the impact of luminal attenuation on plaques and found that within a 2-pixel radius outside the lumen, the influence followed an exponential decay pattern: *y* = *A*e^−*λx*^ + *c* (where *A*, *λ*, *x*, and *c* represent amplitude, coefficient, distance from the luminal boundary, and constant, respectively). They recommended selecting ROIs at a distance of at least 0.8 mm from the lumen to minimize the partial volume effect, providing insights for accurate plaque measurement. However, this approach has limitations in reflecting the overall plaque nature and volume of individual components, especially for small-volume plaques.

Secondly, low tube voltage is a primary means of reducing radiation dose in CCTA. However, due to the weak penetration ability of low-energy x-rays, it significantly influences plaque assessment based on CT values (22). Wang et al. (15) compared the effects of conventional and low tube voltage on the HU of non-calcified plaques using a phantom model. The results showed that the HU of low tube voltage plaques were significantly higher than those at conventional voltage. Different tube voltages require different cutoff HU for distinguishing lipid and fibrous components. For example, the volumes of both components measured at 80 kV (82 HU) and 120 kV (60 HU) showed good correlation (*r* = 0.841, *r* = 0.879). The study indicated two pathways through which tube voltage influences plaques: the direct pathway driven by x-ray energy and the indirect pathway by altering luminal attenuation, reducing tube voltage would decrease the Compton effect, raising the HU of the iodine-containing contrast agent (23). Matsumoto et al. (11) compared the effects of 100 kV and 120 kV on plaque components, finding that lower tube voltage led to a significant increase in the HU of fibrous and lipid components, by approximately 2.2 HU and 5.3 HU, respectively. However, after correcting for the indirect effect of luminal attenuation, 100 kV had almost no direct impact on non-calcified plaques. Takagi et al. (20) found that compared to 120 kV, 100 kV increased calcium volume (2.4%) but decreased fibrofatty (4.8%) and necrotic core (1.5%) volumes. This change was primarily induced by the indirect pathway. Besides the indirect pathway, the authors further discovered that even lower tube voltage (80 kV) directly affected fibrofatty and necrotic core components, increasing their volumes by 6.2% and 0.9%, respectively. However, both 100 kV and 80 kV had a direct impact on calcified components, possibly due to the higher likelihood of photoelectric interaction in dense tissue at lower x-ray energies, with the maximum variation occurring in high-density calcium.

Apart from luminal attenuation and tube voltage, the accuracy of plaque evaluation is also influenced by scanning parameters, post-processing methods, and subjectivity. Different CT machine models may result in different cutoff HU for plaque assessment (24). The HU of plaques decrease with an increase in detector rows and reach their highest point at maximum collimation width, possibly due to higher spatial resolution reducing partial volume effect. Additionally, using different reconstruction parameters, such as slice thickness and kernel, affects plaque HU. Cademartiri et al. (25) comparing four different convolution kernels (b30f, b36f, b46f, and b60f), found that using sharper kernels increased spatial resolution and image noise, raising the HU of calcified plaques (740.5, 758.5, 785.7, and 1,145.8 HU) and decreasing non-calcified plaque HU (20.8, 14.2, 14.0, and 3.2 HU). Thicker slice thickness also decreases spatial resolution, increasing plaque HU (26). Moreover, human interpretation is prone to fatigue and subjectivity. In many CTA studies, plaque CT values may be influenced by the selection, position, and size of ROIs (27).

### 1.3 Improvement methods

In addressing the aforementioned influencing factors, multiple researchers emphasize that achieving similar luminal attenuation by unifying imaging parameters may be the most ideal solution, especially in the context of serial CCTA examinations within the same patient cohort (11, 20). However, the complete standardization of parameters to eliminate variations posed by CT machine models, reconstruction methods, physician experience, and individual patient differences is challenging. In light of this, various studies have proposed adaptive approaches, dual-energy techniques, dose adjustments of contrast agents, and artificial intelligence (AI) methods as potential solutions (3, 11, 28–38).

Firstly, to mitigate the impact on luminal attenuation, de Knegt et al. (28) proposed an adaptive method by adjusting plaque assessment thresholds based on luminal CT values. Results indicated that adaptive thresholds more accurately assessed plaques, whereas conventional CT values underestimated the volume of fibrous and fibrofatty plaques and overestimated the volume of necrotic cores and calcifications. Another study utilized the average luminal CT values of plaques in the proximal and distal regions for calibration, revealing an overlap in the conventional CT values distributions of lipid and fibrous plaques (−4.5 to 74.6 vs. 61.9–149.7). However, standardized CT values (plaque/luminal) ratios eliminated this overlap (−0.012 to 0.147 vs. 0.153–0.394) (11). Nonetheless, luminal CT values gradually decrease with increasing stenosis severity and decreasing diameter, leading to a significant reduction in luminal CT values in severely stenosed regions (29). Therefore, even with adjustments for scan-to-scan variations in luminal attenuation, applying the same thresholds in stenotic and non-stenotic regions may still be inadequate. Shin et al. (30) proposed two novel adaptive thresholds: scan-adaptive threshold, using the same threshold at each cross-section, and position-adaptive threshold, applying different CT thresholds at each cross-section (i.e., <50% * corresponding cross-sectional luminal CT value for non-calcified plaques, >110% * corresponding luminal CT value for calcified plaques). The results indicated that scan-adaptive thresholds overestimated plaque volume, while position-adaptive thresholds exhibited higher accuracy.

In addition, dual-energy CT (DECT) can optimize image quality based on characteristic energy levels, distinguishing tissue types by absorbing high and low-energy x-rays. DECT provides additional parameters such as iodine maps, effective atomic number maps, and virtual monoenergetic images, yielding more diagnostic information. Recent research suggests that non-calcified plaques can be accurately identified using effective atomic number maps (32). Although multi-energy CT has advantages in plaque characterization, it faces limitations related to the complexity of plaque composition and the small size of coronary artery plaques, posing challenges for quantitative analysis due to partial volume effects (39).

Secondly, addressing the impact of tube voltage, adjusting the corresponding contrast agent dose can alleviate changes inluminal attenuation and improve the qualitative and quantitative assessment of plaques. Yin et al. (33) found that CCTA images and luminal attenuation obtained with 100 kV combined with lower concentration iodine contrast (270 mg iodine/ml) were similar to those obtained with 120 kV combined with higher concentration contrast (370 mg iodine/ml). Jia et al. (34) discovered that images obtained with 70 kVp combined with 30 ml of lower concentration contrast (300 mg iodine/ml) were comparable in quality to those obtained with 100/120 kVp combined with 65–75 ml of the same concentration contrast. These studies suggest that low-dose scans based on low tube voltage can reduce radiation dose while avoiding excessive luminal attenuation. However, this approach is associated with higher image noise and lower contrast-to-noise ratio, impacting accurate plaque quantification and characterization. Iterative reconstruction (IR) combined with low tube voltage scans is beneficial for maintaining diagnostic image quality while reducing radiation dose. Studies indicate that deep learning-based image reconstruction algorithms can reduce radiation dose by over 40% and improve image quality by 62%, without affecting stenosis severity, plaque composition, and quantitative assessment (35). However, there is limited literature on directly addressing the impact of low tube voltage, necessitating further research.

To mitigate subjective influences, studies have found that histogram analysis and AI-based algorithms can overcome the subjectivity of ROI selection by analyzing the entire plaque. Matsumoto et al. (3) utilized histogram analysis with IVUS as the gold standard, revealing that the area under the curve for diagnosing lipid-rich plaques based on the proportion of CT values ≤30 HU was significantly higher (0.9) than that for conventional CT values (0.3), with sensitivity and specificity reaching 95% and 80%, respectively. While histogram analysis eliminates the subjective impact of ROI selection, it does not account for objective influences such as luminal attenuation, resulting in varying threshold values across different studies (36). Additionally, AI-based processing algorithms can achieve image reconstruction, segmentation, measurement, and feature extraction with high accuracy and low reader variability, potentially reducing subjective influences while mitigating objective influences such as luminal attenuation (36, 37). Kolossváry et al. (38) processed CT images of 44 coronary artery plaques, extracting radiomic features (935 in total). The results indicated that, using NaF 18-positron emission tomography, intravascular ultrasound, and optical coherence tomography as gold standards, the diagnostic value of radiomic features in identifying vulnerable plaques was significantly superior to conventional CT parameters, with increased areas under the curve of 0.22, 0.13, and 0.14, respectively. However, whether radiomic features can classify plaque components and their capabilities remain unreported and warrant further investigation.

## 2 Conclusion

While coronary computed tomography angiography (CCTA) has become a common non-invasive imaging method for evaluating plaques, it can not only reliably evaluate luminal stenosis and its functional significance, but also accurately evaluate the morphology and composition of plaque and identify high-risk plaque, it is of great significance to guide the clinical management of patients with coronary heart disease, accurate evaluation of plaques based on CCTA still faces numerous challenges due to factors such as luminal attenuation, parameter settings, and subjectivity. The use of standardized scanning methods, self-adaption, dual-energy techniques, and artificial intelligence (AI) holds promise in addressing these challenges. Particularly, AI has demonstrated significant advantages in enhancing image quality and deeply exploring plaque characteristics, showing considerable potential for clinical applications. However, further research is needed to address the direct impact of improving low tube voltage.
